# Liraglutide modulates cyclooxygenase and α7 acetylcholine receptors: in vitro and in silico insights into its anti-inflammatory role in LPS-induced inflammation in RAW 264.7 macrophages

**DOI:** 10.1007/s00210-025-04225-5

**Published:** 2025-05-31

**Authors:** Elif Baris, Huseyin Saygin Portakal, Arda Aslan, Zeynep Firtina Karagonlar, Metiner Tosun

**Affiliations:** 1 Department of Medical Pharmacology, Faculty of Medicine, Izmir University of Economics, Sakarya Cd. 156, 35330 Izmir, Türkiye; 2 Department of Genetics and Bioengineering, Faculty of Engineering, Izmir University of Economics, Izmir, Türkiye

**Keywords:** Liraglutide, Prostaglandins, Inflammation, Cyclooxygenase, Cholinergic, Nicotinic, In silico analysis

## Abstract

Liraglutide, a glucagon-like peptide-1 (GLP-1) receptor agonist, is well-established for its metabolic benefits, including glycemic control and weight loss. Beyond these roles, it exhibits significant anti-inflammatory properties, though the mechanisms remain underexplored. This study investigates the anti-inflammatory effects of liraglutide in lipopolysaccharide (LPS)-stimulated RAW 264.7 murine macrophages. Results demonstrate that increasing concentrations of liraglutide suppresses LPS-elevated prostaglandin E2 (PGE2), 6-keto prostaglandin F1α (6-keto-PGF_1α_, a stable prostacyclin metabolite) and thromboxane A2 (TXA2), similar to that observed with conventional anti-inflammatory agents, ibuprofen and celecoxib. Mechanistic exploration reveals that liraglutide's anti-inflammatory action is dually-modulated by cyclooxygenase (COX) and nicotinic acetylcholine receptor (nAChR) signaling. The application of non-selective, non-competitive nAChR antagonist or selective and potent α7-nAChR antagonist, mecamylamine (MEC) and methyllycaconitine (MLA), respectively, highlights the involvement of cholinergic pathways in liraglutide's activity. Based on in silico molecular docking analyses, liraglutide exhibits favorable binding affinities to COX-1, COX-2, prostacyclin synthase (PGIS), and α7nAChRs, supporting its potential multi-target anti-inflammatory effects. These findings suggest that the therapeutic potential of liraglutide may go beyond metabolic regulation and may be promising for conditions in which metabolic and inflammatory pathways converge, including inflammation and modulation of cholinergic signaling.

## Introduction

Liraglutide, a glucagon-like peptide-1 (GLP-1) receptor agonist, is widely used in the treatment of type 2 diabetes mellitus and obesity. Its clinical benefits include significant reductions in HbA1c levels and weight loss. Beyond its metabolic applications, liraglutide has been shown to reduce oxidative stress and promote neuronal recovery in hypoxic-ischemic injury models in neonatal rats, as well as decrease hepatic inflammation in non-alcoholic steatohepatitis models, highlighting its anti-inflammatory potential (Ipsen et al. 2018; Zeng et al. 2019). These findings support studies demonstrating liraglutide's capacity to reduce atherosclerosis by modulating inflammatory pathway and reinforce its cardiovascular benefits (Rakipovski et al. 2018).

Liraglutide's interaction with the cholinergic system has garnered interest due to its implications in both metabolic regulation and cognitive function. Accumulated data suggest that, in addition to enhancing insulin secretion, liraglutide improves cognition and working memory in rats with drug-induced cognitive deficits (Babic et al. 2018). These effects may also be related to liraglutide’s interaction with cholinergic receptors, particularly α7 nicotinic acetylcholine receptors (α7nAChRs) which are implicated in both cognitive and autonomic functions. This interaction may extend to its effects on GI motility, as GLP-1 receptor signaling has been implicated in modulating GI contractions potentially via central cholinergic pathways. Such modulation is particularly relevant for diabetic patients who often experience altered gastrointestinal (GI) motility due to neuropathy (Faillie et al. 2022). Liraglutide is often associated with a range of GI side effects such as nausea, vomiting, diarrhea, and abdominal discomfort. Nausea is observed up to 67% of patients during treatment, a substantially higher incidence compared to 26% in placebo groups (Kim et al. 2014). Although generally transient, severe GI adverse reactions commonly lead to compliance problems in patients (Jiang et al. 2023). Diarrhea is also prevalent but decreases with gradual dose adjustments (Pi-Sunyer et al. 2015; Sanyal and Majumdar 2013). In rarer instances, liraglutide delayed gastric emptying, contributing to gastroparesis and complicating diabetes management (Ishihara et al. 2022). Rarely, concerns have also been raised about potential risks of pancreatitis and thyroid cancer with long-term use (Fan et al. 2018). Gallbladder issues, particularly gallstone formation in patients experiencing rapid weight loss, have also been documented. While severe hypoglycemia is uncommon, careful monitoring is recommended when liraglutide is combined with other antidiabetic drugs (Jiang et al. 2023; Pi-Sunyer et al. 2015; Pratley et al. 2011). Notably, several of these effects—such as GI disturbances, and autonomic dysfunction—appear to be associated with disruptions in cholinergic signaling, all of which emphasize liraglutide’s potential cholinergic interactions.

Accumulated data suggest that liraglutide may act through both central and peripheral mechanisms, modulating the cholinergic anti-inflammatory pathway (CAP). Hence, GLP-1 receptor agonists may interact with α7nAChRs, a key nicotinic receptor subtype involved in cholinergic anti-inflammatory signaling. LPS-induced inflammation in RAW 264.7 macrophages is a well-established model that mimics innate immune activation via Toll-like receptor 4 (TLR4) signaling, leading to the release of pro-inflammatory mediators such as cytokines and prostaglandins. This makes it a widely accepted and reproducible system to investigate the prostaglandin mediated anti-inflammatory potential of candidate compounds like liraglutide. Therefore, this study investigates the prostaglandin-mediated effects of liraglutide in an LPS-induced in vitro inflammation model employing RAW 264.7 macrophages as well as potential interactions with the COX pathway and cholinergic receptors in silico.

## Materials and methods

### Chemicals

Liraglutide (Saxenda®) was purchased from Novo Nordisk (Batch number: PZF8 K92) and Lipopolysaccharide (LPS, from Escherichia coli O111:B4) from Sigma-Aldrich (L2630). Ibuprofen (Santa Cruz, sc-200534) and celecoxib (MedChemExpress, HY-14398) were used as COX pathway modulators. Mecamylamine hydrochloride (MEC, Sigma-Aldrich, M9020), a non-selective nicotinic acetylcholine receptor (nAChR) antagonist, and methyllycaconitine citrate (MLA, Sigma-Aldrich, M168), a selective α7 nAChR antagonist, were used for receptor-specific inhibition studies. All chemicals were dissolved in sterile phosphate-buffered saline (PBS, pH: 7.4; Capricorn, PBS-1 A) to prepare stock solutions and diluted with culture media immediately before each experiment.

### Cell culture

RAW 264.7 macrophages at passage #10 (ATCC TIB 202, Manassas, VA) were maintained in DMEM (Sigma Aldrich D6429), supplemented with heat-inactivated FBS (10%) and penicillin (100 U/mL), streptomycin (100 μg/mL, Gibco, Carlsbad, CA) at 37 °C in a CO_2_ (5%) incubator. Cells (1,000,000/well) were seeded in 6 well tissue culture plates after collecting. Before the administration of the drugs, the medium was replaced with fresh media for all treatment groups.

In the first set of experiments, cells were exposed to a range of liraglutide concentrations (1–500 nM) to evaluate cell viability and establish the non-toxic concentration range. The cell viability assay employed the reduction of the yellow substrate, 3-(4,5-dimethylthiazol-2-yl)−2,5-diphenyltetrazolium bromide (MTT), into purple formazan by metabolically active mitochondria. (Elabscience, E-CK-A341) (Meurot et al. 2022). In the second set, cells were treated with increasing concentrations of liraglutide (1–25 nM, non-cytotoxic range) 30 min prior to LPS administration 1 μg/mL) to determine effective concentration on LPS-induced PG levels (Baris et al. 2023). In the third set, the effect of liraglutide (10nM) was compared with that of ibuprofen (0.5 µM,) and celecoxib (3 µM,) (Sivalingam et al. 2021; Jeon et al. 2000). In the fourth set, to investigate the involvement of nAChRs, a non-selective nAChR antagonist mecamylamine hydrochloride (MEC, 50 μΜ,) and selective α7nAChR antagonist methyllycaconitine citrate (MLA, 1 μΜ,) were applied 30 min before liraglutide (10nM) and LPS (Baris et al. 2021; Yang et al. 2015; Yi et al. 2015). All in vitro experiments were performed in six biological replicates (n = 6). The control group composed of untreated RAW 264.7 macrophages maintained in complete culture media without LPS or liraglutide exposure.

### Protein analyses

Commercial enzyme-linked immunosorbent assay (ELISA) kits were used to quantify PGE2 (Elabscience-E-EL-0034), 6-keto prostaglandin F1α (a stable analog of PGI2, Elabscience E-EL-0054) and Thromboxane A2 (TXA2, Elabscience E-EL-0057) levels released into the culture media 24 h after LPS administration according to the manufacturer’s instructions. Absorbance was measured at 450 nm via BioTek ELx800 microplate reader.

### In silico analyses

Protein–protein docking is a widely used molecular modelling approach to predict binding affinity of a protein to a potential partner and their interactions within protein interfaces considering steric and physicochemical complementarity at the protein–protein interface. In order to predict potent binding affinity of Liraglutide and GLP-1 to candidate partners, both peptides were docked to α7nAChR, COX-2, and prostacyclin synthase (PGIS) proteins’ previously identified binding regions by their inhibitors via HADDOCK 2.4. protein–protein docking server. Initially, crystal structures of α7nAChR, COX-2, PGIS, Liraglutide, and GLP-1 were downloaded from Protein DataBank (PDB IDs: 7EKP, 3LN1, 3B6H, 4 APD, and 3IOL, respectively). Binding regions of all proteins were isolated from the structures and prepared for further protein-peptide docking application using 1.16 version of UCSF Chimera software’s Dock Prep module by removing water, heteroatoms, and ligands, adding hydrogen atoms and partial charges, and replacing the side chains. Amino acid residues of α7nAChR, COX-2, and PGIS recognized by the respective inhibitors, I33, celecoxib, and minoxidil which are found in the retrieved crystal structures were analyzed and utilized as restraining inputs for protein-peptide docking in HADDOCK 2.4. server. Lastly, the obtained interactions within protein interfaces of protein-peptide models were analyzed with PyMol software (Zundert and Bonvin 2014).

### Statistics

**Shapiro–Wilk test** was performed to assess the normality of all datasets. One-way analysis of variance analysis (ANOVA) with post*-*hoc Tukey–Kramer multiple comparison tests for multiple comparisons for MTT analyses. ELISA data were analyzed using Mann–Whitney U test (GraphPad Prism 5, La Jolla, CA). Data were expressed as mean ± S.E.M (n = 6, each performed in triplicate) and *p* < 0.05 was accepted as statistically significant.

## Results

### Cytotoxicity of liraglutide on RAW 264.7 macrophage cells

**Fig. 1:**
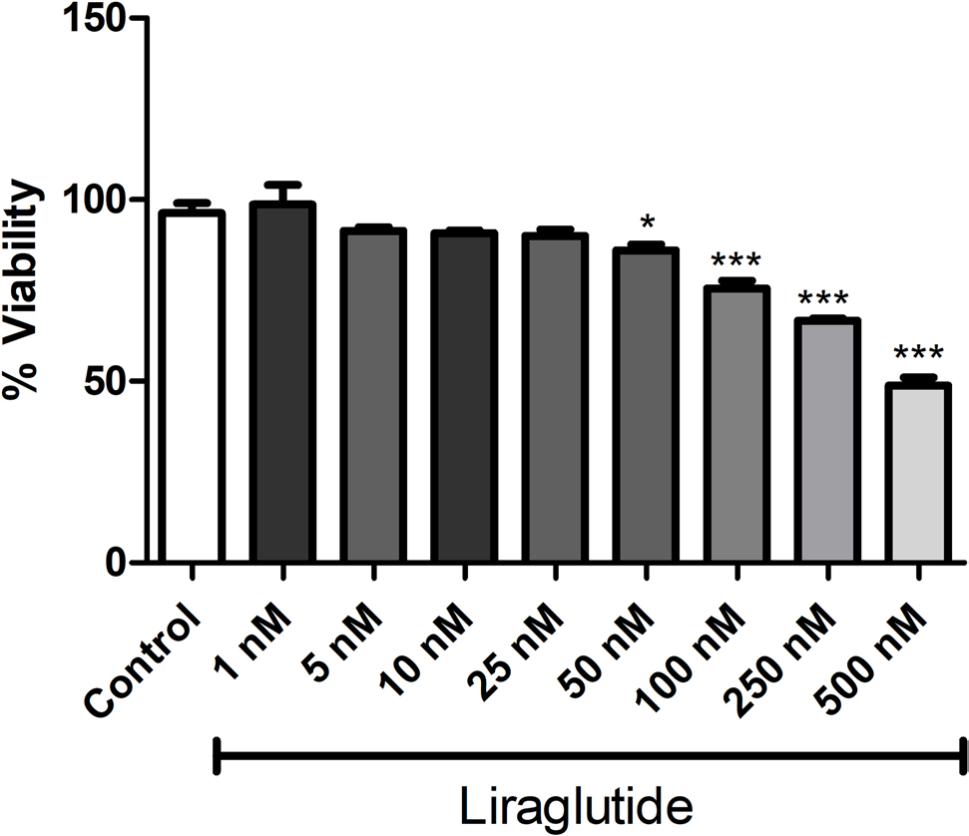
Cytotoxicity assay of liraglutide performed on RAW 264.7 macrophages. Shown are the effects of increasing concentrations of liraglutide on cell viability (% control). Data are shown as mean ± S.E.M. (***, *p* < 0.001 and *, *p* < 0.05 vs control; *n* = 6)

MTT assay results demonstrated that liraglutide at concentrations of 1, 5, 10, and 25 nM did not significantly affect the viability of RAW 264.7 cells compared to control cells containing only culture medium, confirming suitability of these concentrations for subsequent experiments (Fig. 1).

### Inhibitory effects of Liraglutide on LPS-elevated PG levels

**Fig. 2:**
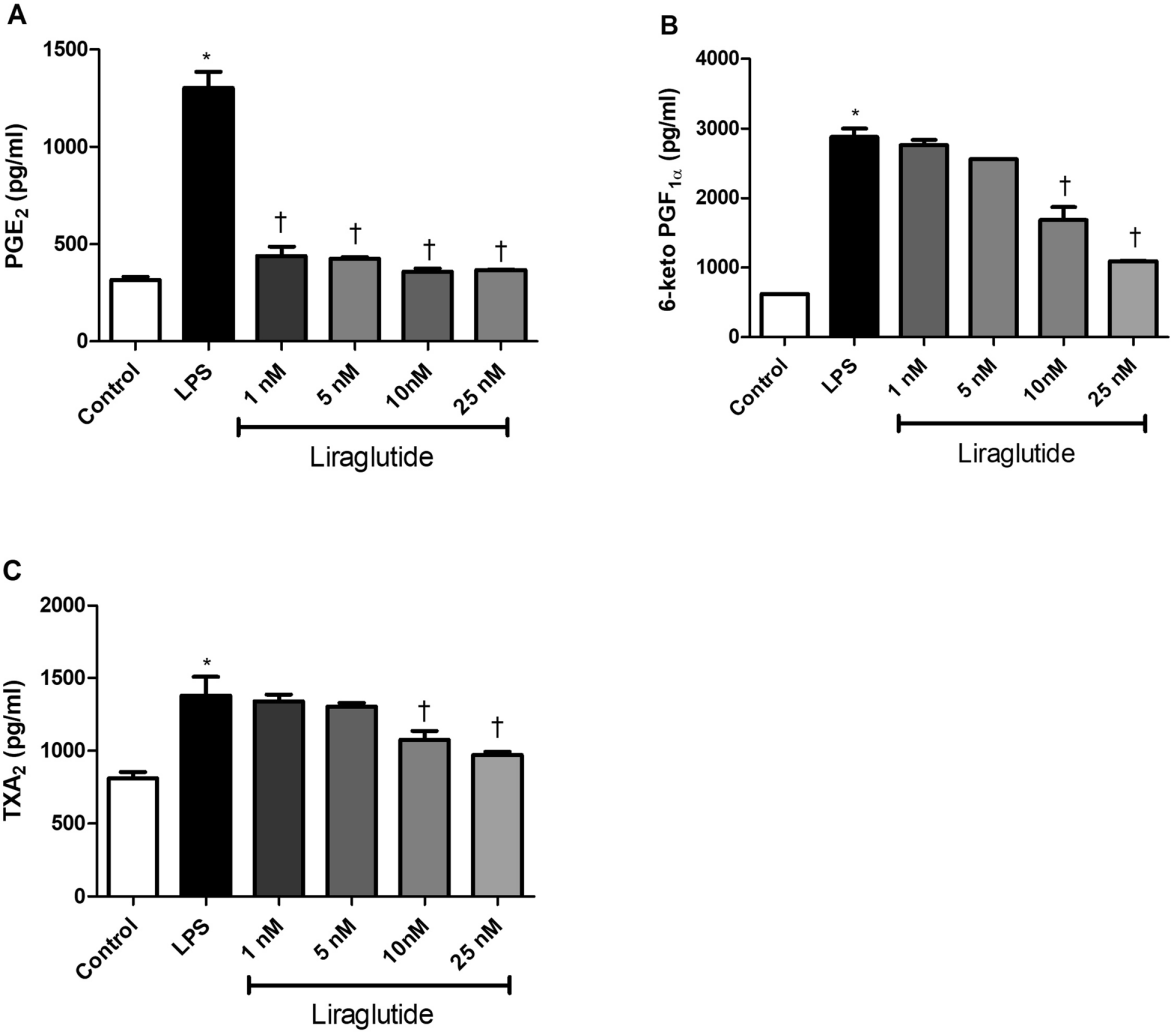
Effects of liraglutide on LPS-induced PG elevations. Shown are the effects of increasing concentrations of liraglutide on LPS-induced prostaglandin E2 (PGE2) (**A**), 6-keto PGF1α (a stable analog of PGI2) (**B**) and Thromboxane (TXA2) (**C**) levels. Data are shown as mean ± S.E.M. (*, *p* < 0.05 vs control; †, *p* < 0.05, vs LPS, *n* = 6). LPS: Lipopolysaccharide

**Fig. 3:**
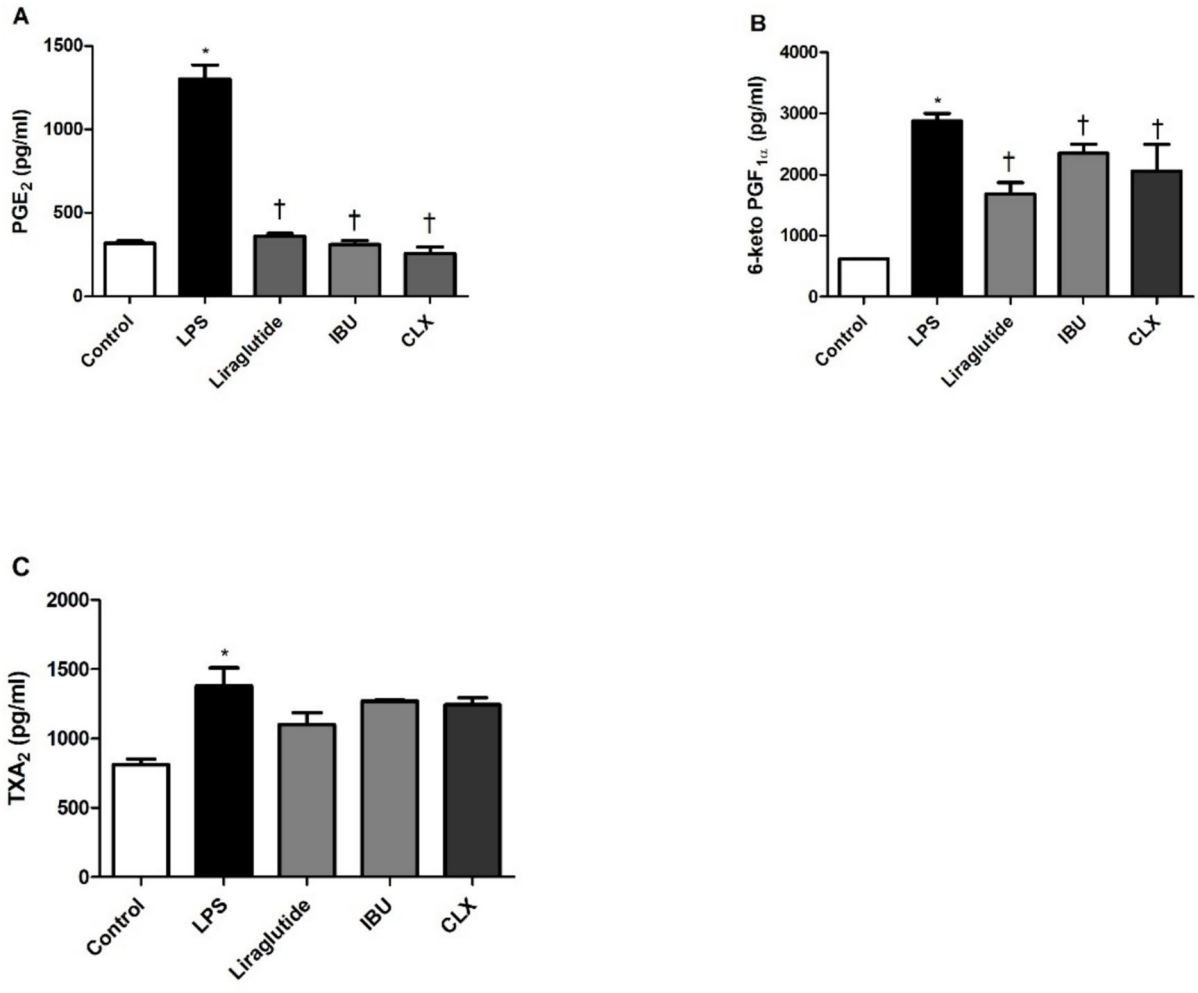
Effects of liraglutide vs ibuprofen and celecoxib on LPS-induced PG elevations. Shown are the effects of liraglutide (10 nM) on 1 µg/ml LPS -induced Prostaglandin E2 (**A**), 6-keto prostaglandin F1α (**B**), Thromboxane A2 (**C**) levels compared to ibuprofen (0.5 µM) and celecoxib (3 µM). Data are shown as mean ± S.E.M. (*, *p* < 0.05 vs control; †, *p* < 0.05 vs LPS, *n* = 6). LPS: Lipopolysaccharide, CLX: Celecoxib, IBU: Ibuprofen

RAW 264.7 cells were stimulated with 1 µg/mL LPS, a concentration previously optimized in our previous studies to induce inflammatory responses without affecting cell viability (Baris et al. 2023, 2024; Baris et al. 2021). Liraglutide suppressed LPS-elevated PGE2 levels (Fig. 2A) whereas only 10nM and 25nM concentrations decreased 6-keto PGF1α (Fig. 2B) and TXA2 (Fig. 2C) levels. LPS-elevated PG levels were also suppressed by ibuprofen, and celecoxib (Fig. 3) similar to that of 10 nM liraglutide. PG levels were not altered by drug treatment or DMSO *per se* (not shown).

### nAChR-mediated suppression of LPS-induced PG elevations by liraglutide

**Fig. 4:**
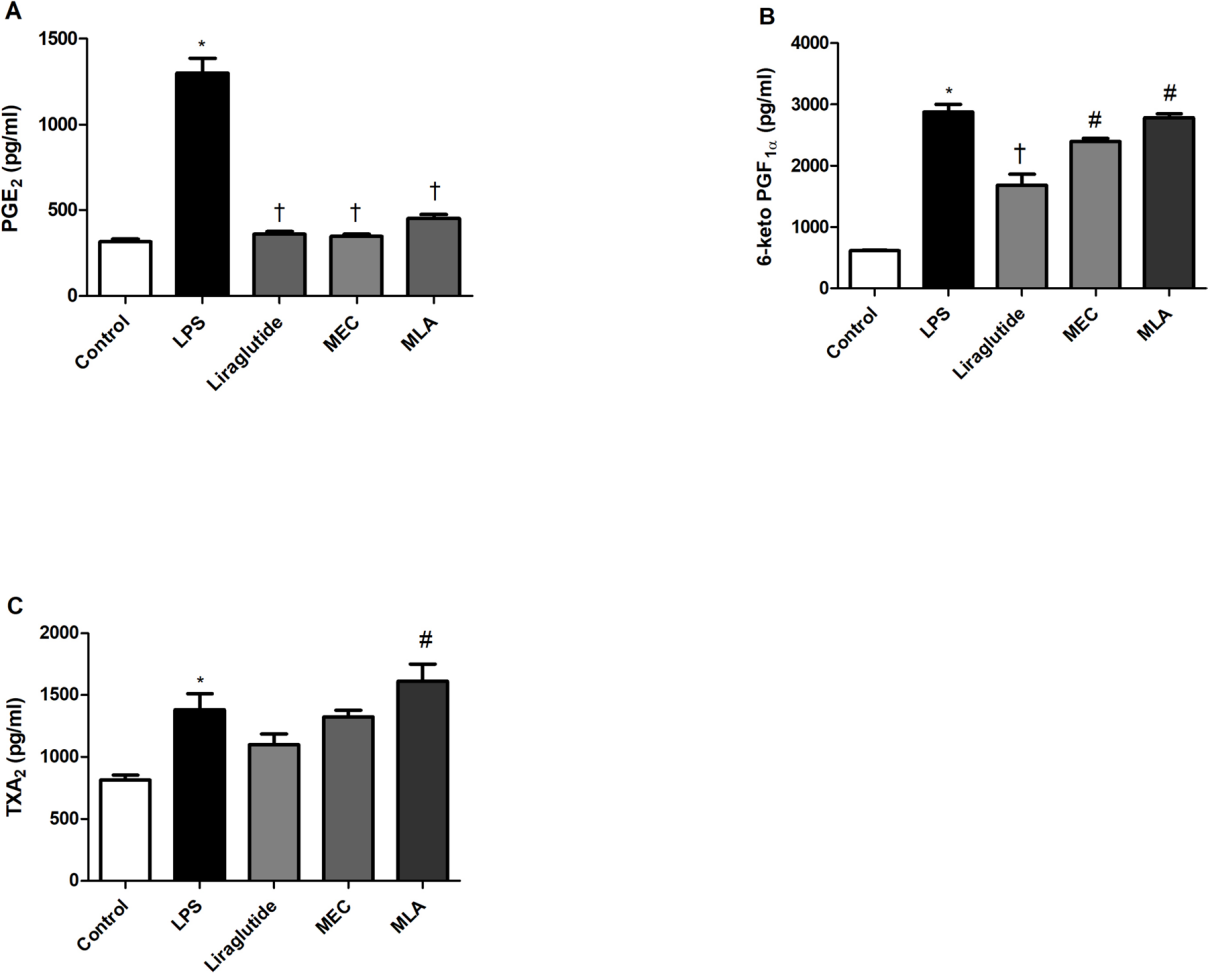
Effects of Liraglutide on LPS-induced PG elevations in the presence and absence of non-selective nAChR or α7nAChR antagonists. Shown are the effects of liraglutide (10 nM) on 1 µg/ml LPS -induced Prostaglandin E2 (**A**), 6-keto prostaglandin F1α (**B**), Thromboxane A2 (**C**) levels with or without mecamylamine (MEC, 50 μΜ) or methyllycaconitine (MLA, 1 μΜ). Data are shown as mean ± S.E.M. (*, *p* < 0.05 vs control; †, *p* < 0.05 vs LPS group; ‡, *p* < 0.05, vs LPS + VAR, *n* = 6). LPS: Lipopolysaccharide, MEC: mecamylamine, MLA: methyllycaconitine

RAW 264.7 cells were pretreated with mecamylamine (MEC) and/or methyllycaconitine citrate (MLA) prior to the incubation with LPS (1 µg/ml) and liraglutide (10 nM) for 24 h. 6-keto PGF1α and TXA2 levels were increased in MEC and MLA groups compared to liraglutide-treated groups. In the pre-treated groups, inhibitory effect of liraglutide on 6-keto PGF1α and TXA2 levels was partially reversed suggesting a role for α7nAChR signalling in mediating liraglutide’s action (Fig. 4).

### In silico analyses: modelling of protein-peptide complexes via HADDOCK 2.4. server

The possible interactions of liraglutide and GLP-1 with α7nAChR, COX-2, and PGIS have been modelled using protein-peptide docking approach via HADDOCK 2.4. protein–protein docking server. In order to reveal the interacting regions of the proteins, protein structures were downloaded from PDB databank and the amino acids interacting with the inhibitors have been determined on the crystal structures. Table 1 includes the PDB information, inhibitor names and the interacting amino acids of the proteins. After determining the interacting amino acids, crystal structures of proteins as well as liraglutide and GLP-1 were prepared with Dock Prep module of UCSF Chimera for the protein-peptide docking study. Prepared proteins and liraglutide peptide were then imported to HADDOCK 2.4. protein–protein docking server and the interacting amino acids of the proteins were utilized to define target regions. Table 2 demonstrates results including HADDOCK score, Z-score, and interacting amino acids of each protein with Liraglutide and GLP-1.

**Table 1:** Amino acids of receptors and enzymes interacting with specific ligands identified in.pdb files

Receptor	PDB ID	Ligand	Interacting amino acids
α7nAChR	7EKP	I33	TRP77, LEU141, TRP171, TYR210, CYS212, TYR217
COX-2	3LN1	Celecoxib	ARG106, GLN178, VAL335, LEU338, SER339, LEU370, TYR371, TRP373, VAL509, GLY512, ALA513, LEU517
Prostacyclin Synthase (PGIS)	3B6H	Minoxidil	TYR99, ALA100, LRU103, LYS121, MET124, LEU128, LEU180, ALA283, MET288, ASN287, ALA291, PRO355, GLY435, TRP434, HIS440, CYS441, LEU442, ALA447, LEU485

**Table 2:** Protein-peptide docking results including HADDOCK Score, Z-Score and interacting amino acids of the receptors and enzymes with liraglutide and GLP-1

Ligand	Receptor	HADDOCK Score	Z-Score	Interacting amino acids
Liraglutide	α7nAChR	−121.8 ± 18.5	−1.9	ASP104, ASP123, ASN129, SER172, GLU211
	COX-2	−93.7 ± 4.3	−1.6	LYS82, PRO177, ASP333, HIS342, THR547, SER565, ASN567, GLN569
	Prostacyclin Synthase (PGIS)	−136.9 ± 7.7	−1.8	HIS114, SER118, ASP119, ARG123, LYS125, LYS428, TRP434, ARG444
GLP-1	α7nAChR	−105.4 ± 3.3	−2.3	ILE112, ASP123, HIS127, ASN129, TRP171, GLU215, TYR217
	COX-2	−86.8 ± 1.5	−1.7	THR79, LYS82, PRO117, HIS342, ASP501
	Prostacyclin Synthase (PGIS)	−125.2 ± 11.4	−2.4	ASP119, ARG123, LYS125, ASP342, LYS418, ASN431, HIS438, ARG444

The HADDOCK scores indicating the energy (Delta G free energy) units to create the interactions between biomolecules, reveal that liraglutide has potential high binding affinity to α7nAChR, COX-2, and PGIS since the HADDOCK scores of models are −121.8 ± 18.5, −93.7 ± 4.3, and −136.9 ± 7.7, respectively. In addition, The HADDOCK Scores of GLP-1 to α7nAChR, COX-2, and PGIS were also similar to Liraglutide with scores of −105.4 ± 3.3, −86.8 ± 1.5, and −125.2 ± 11.4, respectively.

**Fig. 5:**
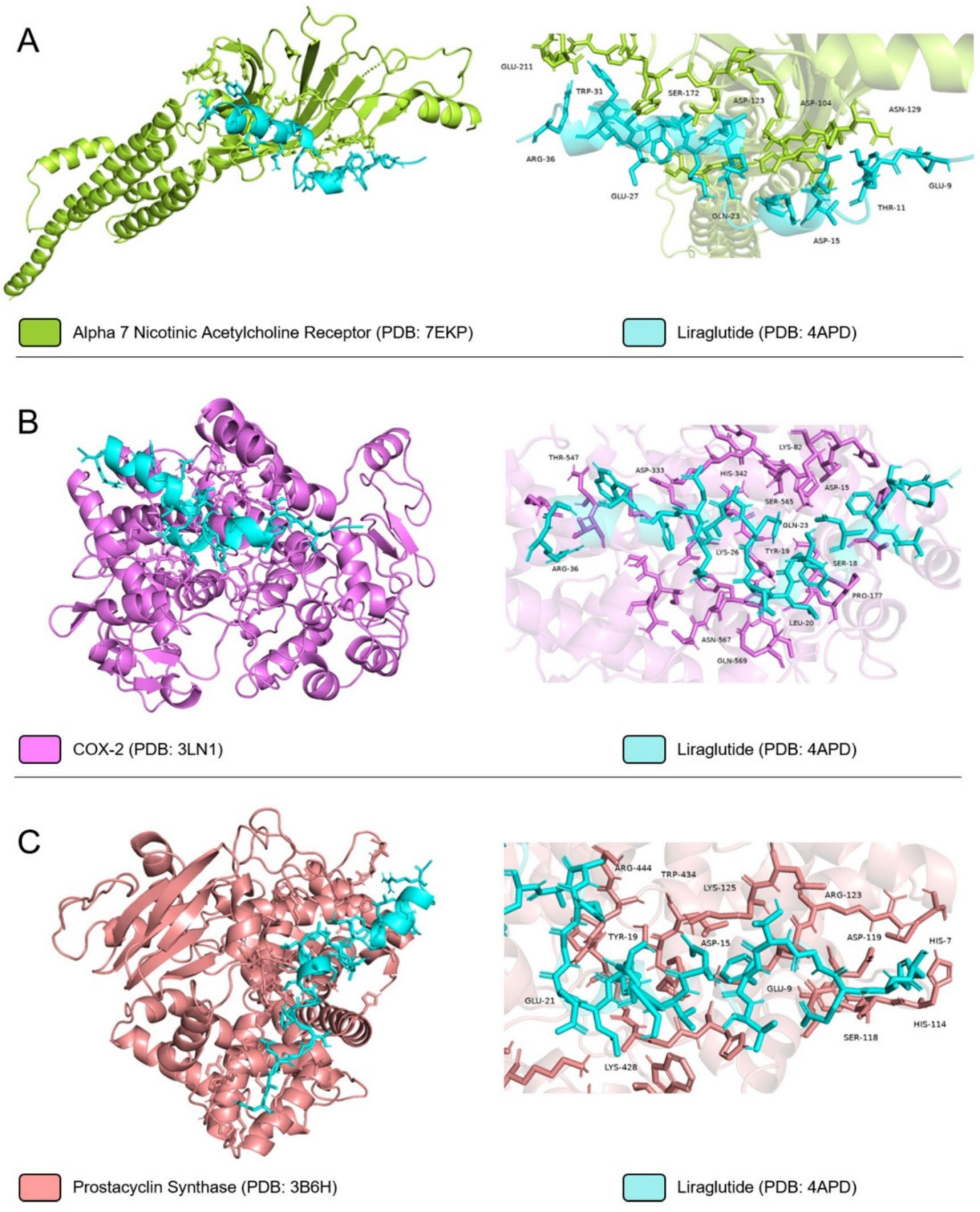
Predicted interactions of liraglutide (PDB: 4 APD) with (**A**) α7nAChR (PDB: 7EKP), **B** COX-2 (PDB: 3LN1), and **C** Prostacyclin Synthase (PDB: 3B6H) modeled with HADDOCK 2.4. protein–protein docking server

**Fig. 6:**
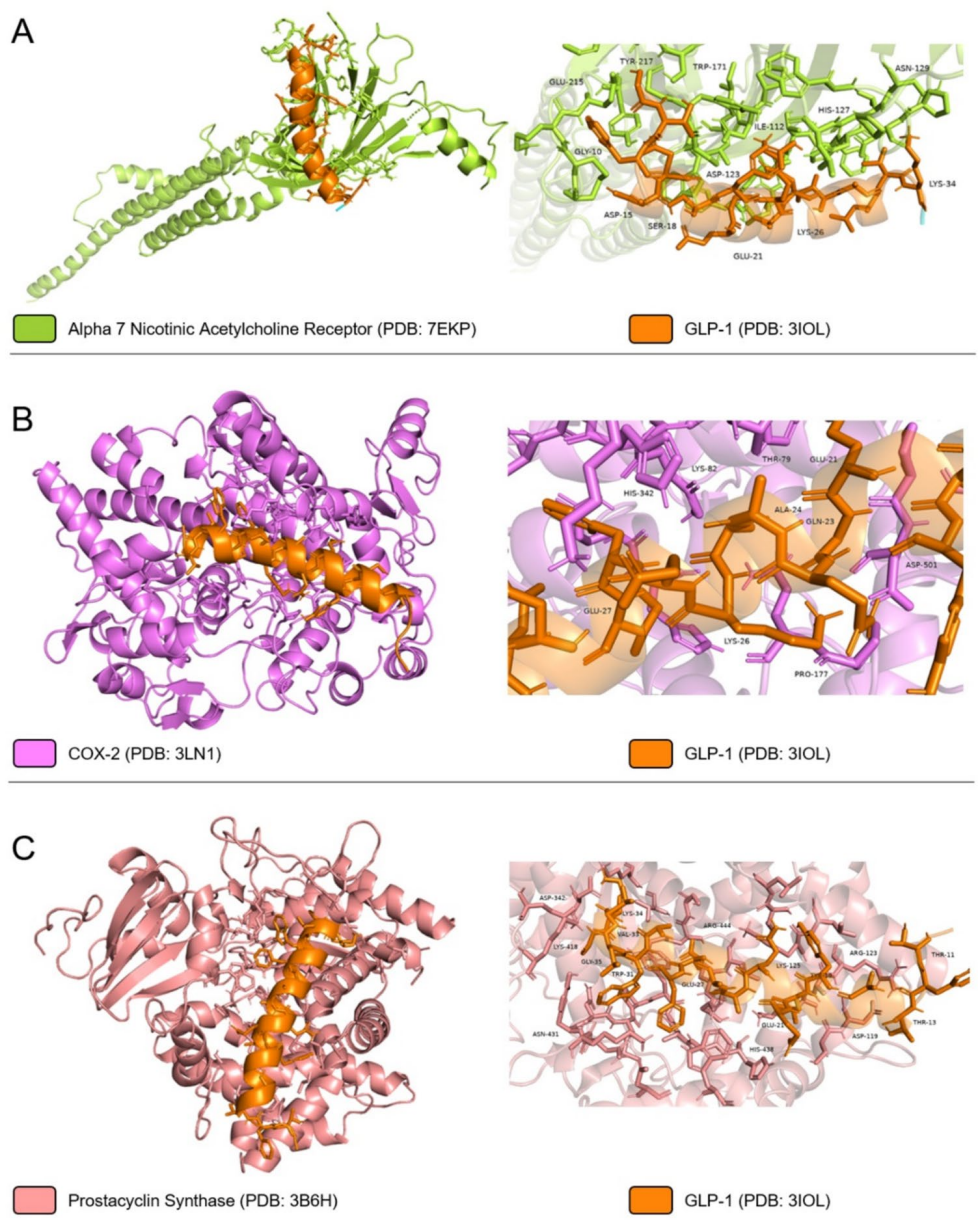
Predicted interactions of GLP-1 (PDB: 3IOL) with (**A**) Alpha 7 Nicotinic Acetylcholine Receptor (PDB: 7EKP), **B** COX-2 (PDB: 3LN1), and **C** Prostacyclin Synthase (PDB: 3B6H) modeled with HADDOCK 2.4. protein–protein docking server

The similarity of HADDOCK scores of the models between the protein complexes of liraglutide and GLP-1, suggest that the binding affinity of liraglutide is similar to GLP-1’s affinity for all candidate proteins analyzed. Furthermore, the highly negative Z-scores indicate the reliability of the data, confirming that the models were generated with sufficient efficiency. In addition, the clusters created with HADDOCK server were analyzed in PyMol software and the interactions found within the interface of proteins interacting with liraglutide and GLP-1 were analyzed. The results show that liraglutide possibly interacts with ASP104, ASP123, ASN129, SER172 and GLU211 amino acids of Alpha-7; LYS82, PRO177, ASP333, HIS342, THR547, SER565, ASN567 and GLN569 amino acids of COX-2, and HIS114, SER118, ASP119, ARG123, LYS125, LYS428, TRP434 and ARG444 amino acids of PGIS (Table 2, Fig. 5). In addition, GLP-1 possibly interacts with ILE112, ASP123, HIS127, ASN129, TRP171, GLU215, and TYR217 amino acids of α7nAChR; THR79, LYS82, PRO117, HIS342 and ASP501 amino acids of COX-2, and ASP119, ARG123, LYS125, ASP342, LYS418, ASN431, HIS438 and ARG444 amino acids of PGIS (Table 2, Fig. 6). Regarding the interacting amino acids of proteins with the inhibitors, liraglutide and GLP-1 exhibit similarity indicating the efficiency of the target region design and protein-peptide docking strategy.

## Discussion

In this study, we demonstrated that liraglutide significantly reduced the levels of (PGE2), (6-keto PGF_1α_, a stable prostacyclin PGI_2_ metabolite), and (TXA2) in (LPS)-stimulated RAW 264.7 macrophages, supporting its potential role in modulating inflammatory mediators via the cyclooxyganese (COX) pathway. Prostaglandins (PG) are key lipid-derived mediators involved in classical signs of inflammation, such as vasodilation, vascular permeability, and nociceptor sensitization (Ricciotti and FitzGerald 2011). The observed reduction in PG levels may contribute to the anti-inflammatory properties of liraglutide, in line with previous findings showing that liraglutide reduces proinflammatory cytokine expression (Rakipovski et al. 2018). Our analysis further suggests that liraglutide may interact with key enzymes in the prostaglandin synthesis pathway, including COX isoforms and prostacyclin synthase. Although liraglutide reportedly inhibits TXA2-induced platelet aggregation and reduces vascular inflammation in clinical contexts (Mashayekhi et al. 2023; Ripa et al. 2021), our results offer preliminary mechanistic insight partly underlying these effects. However, as our current findings are limited to in vitro and in silico models, these interpretations should be considered exploratory. Suppression of LPS-induced elevations in PGEα, PGI2, and TXA2 levels, observed across nanomolar concentrations, suggests a concentration-related modulation of prostaglandin production. A similar trend of prostaglandin reduction in comparison with known COX inhibitors such as ibuprofen and celecoxib suggests that liraglutide may act through a comparable mechanism in vitro.

These results support that liraglutide can influence inflammatory signaling pathways, particularly those regulated by COX-2 activation during inflammation (Sivalingam et al. 2021; Jeon et al. 2000). While the clinical relevance of liraglutide as an anti-obesity and anti-diabetic agent has been established, its potential contribution to the modulation of inflammatory mediators in obesity-related inflammation requires further investigation in vivo and in clinical models.

Our findings may also have implications for vascular regulation. Previous research has shown that liraglutide's vasodilatory effects may involve PGI2, nitric oxide (NO), and reactive oxygen species (ROS) in vascular tissues (Sélley et al. 2015) While our study did not investigate vascular function directly, the modulation of PGI2 levels in RAW 264.7 cells may reflect one mechanism through which liraglutide contributes to anti-inflammatory processes relevant to cardiovascular health. Although the potential effect of liraglutide on prostaglandin pathways has been associated with reduced atherosclerotic progression in preclinical models (Yue et al. 2019), further studies are needed to confirm whether these effects are through direct modulation of COX or in vivo (PGIS).

PGI2 plays a known role in vascular homeostasis, with its variable effects by age and disease state.(Vanhoutte et al. 2009). While our study did not focus on vascular consequences, the suppression of PGI2 by liraglutide observed in macrophages suggests its broader potential in modulating inflammation-related prostaglandin signaling. Notably, our in silico findings suggest a potential interaction between liraglutide and PGIS, which could partially explain the reduction in PGI2 observed in vitro. Previous studies have reported PGI2’s dual role as a vasodilator or endothelium-derived contracting factor (EDCF), depending on age and disease state (Rapoport and Williams 2024) and liraglutide has been shown to reduce oxidative stress and inflammation in endothelial cells (Tosun and Rapoport 1996; Biesenbach et al. 2023). While our data are limited to a macrophage model, the suppression of PGI2 by liraglutide—comparable to ibuprofen and celecoxib—suggests that this agent may modulate inflammatory lipid mediators through a mechanism involving PGIS. These findings warrant further in vivo investigation of vascular models to determine the extent to which liraglutide's anti-inflammatory effects translate to cardiovascular contexts.

GLP-1 receptor signaling was suggested to modulate GI motility, possibly via central cholinergic pathways (Faillie et al. 2022). These effects, which are relevant to both therapeutic outcomes and known side effects of liraglutide, point to the involvement of a broader cholinergic system in GLP-1–mediated effects. Although we did not investigate GI or central mechanisms directly, our in vitro findings suggest that liraglutide’s anti-inflammatory effects in LPS-stimulated macrophages may be partly mediated by nAChRs as co-treatment with a nonselective nAChR antagonist (mecamylamine) and a selective α7nAChR antagonist (methyllycaconitine) partially reversed the inhibitory effects of liraglutide on prostaglandin production. These observations are in line with previous findings suggesting that liraglutide may influence inflammatory processes through dual modulation of COX and cholinergic systems (Yang et al. 2014). Our protein-peptide docking analysis also showed predicted interactions between liraglutide and α7nAChR, COX-2, and PGIS. GLP-1 also showed a similar estimated interaction profile with PGIS. These data suggest a mechanistic basis for the action of liraglutide involving both prostaglandin biosynthesis and cholinergic signaling, but further in vivo studies are needed to validate these interactions and their physiological relevance.

The α7nAChR is widely recognized for its role in cholinergic signaling and cognitive function, particularly in brain regions such as the hippocampus and prefrontal cortex (Treinin et al. 2017; Zeisel and Costa 2009). Its activation supports synaptic plasticity and neuroprotection and has been explored as a therapeutic target in neurodegenerative conditions involving cholinergic deficits, such as Alzheimer’s disease. GLP-1 receptor activation has been suggested to influence addiction-related behaviors, including nicotine dependence, through interactions with nAChRs (Egecioglu et al. 2013; Vallöf et al. 2016). Preclinical studies have shown that liraglutide and related GLP-1 analogs can reduce nicotine reinforcement, potentially via modulation of dopaminergic signaling in reward-related brain regions such as the ventral tegmental area (VTA) (Liu et al. 2013). Additionally, liraglutide has been shown to activate AMP-activated protein kinase (AMPK) signaling pathways involved in regulating energy balance and may also play a role in drug-seeking behavior (Wang et al. 2024; Seoane-Collazo et al. 2022). While our study did not assess cognitive outcomes or central nervous system function, previous preclinical research has shown that liraglutide may improve memory performance in aged rats, possibly via anti-inflammatory and neuroprotective effects on cholinergic neurons (Wang et al. 2023). This aligns with findings suggesting GLP-1 receptor activation can restore cholinergic neuron activity, particularly in regions of the brain associated with memory retrieval (Babic et al. 2018). In our in vitro model, the use of α7nAChR antagonists attenuated the anti-inflammatory effects of liraglutide on prostaglandin levels, suggesting that α7nAChR may also contribute to liraglutide’s immunomodulatory activity in macrophages. This finding is further supported by our in silico analysis, which predicted strong binding interactions between liraglutide and α7nAChR. Although the link between liraglutide, α7nAChR, and cognitive outcomes remained indirect in our study, these results support the broader hypothesis that α7nAChR is a key mediator in both inflammatory and cholinergic pathways relevant to liraglutide’s mechanism of action.

## Conclusion

This study demonstrates that liraglutide exerts anti-inflammatory effects in LPS-stimulated RAW 264.7 macrophages, possibly through modulation of prostaglandin synthesis and α7 nicotinic acetylcholine receptors (α7nAChRs). Our in vitro findings, supported by in silico analyses, suggest a potential mechanistic basis for the anti-inflammatory effects of liraglutide. While this study provides mechanistic insights into the anti-inflammatory effects of liraglutide using in vitro and in silico approaches, further research is warranted to validate these findings in vivo. Animal models of inflammation and metabolic disease would be valuable for assessing the physiological relevance of liraglutide’s modulation of prostaglandin pathways and α7nAChR signaling.

## Data Availability

All source data for this work (or generated in this study) are available upon reasonable request.
